# Supplementary material for: Synthesis and Characterisation of Novel Bis(diphenylphosphane oxide)methanidoytterbium(III) Complexes
Source: Molecules. 2022 Nov 9;27(22):7704. doi: 10.3390/molecules27227704 (PMC9698226; doi:10.3390/molecules27227704)
Supplement: Supplementary file 1 [file molecules-27-07704-s001.zip › molecules-1996690-supplementary.pdf]

Electronic Supporting Information

Synthesis and characterisation of novel bis(diphenylphosphane oxide)methanidoytterbium(III) complexes

Shalini Rangarajan[1,2,3], Owen Beaumont[1], Maravanji.S. Balakrishna[2,3], Glen B. Deacon[1,3] Victoria Blair[1,3*]

1 School of Chemistry, Monash University, Clayton, Victoria 3800, Australia

2 Department of Chemistry, Indian Institute of Technology Bombay, Mumbai 400076

3 IITB-Monash Research Academy, Powai, Mumbai 400076

Table of contents

1) Synthesis of compound **1** and monitoring of the consumption of reactants by ^{1}H NMR and ^{31}P{^{1}H} NMR spectroscopy — S2 – S6

2) ^{1}H NMR and ^{31}P{^{1}H} NMR spectra at 25 ^{o}C of the reaction mixture, [$YbCp_2$(DME)] and H_2dppmO_2 (1 : 3) showing evolution of H_2 gas in the reaction mixture. — S6 – S7

3) Averaged natural charges of Yb(III) and OPCPO for **[Yb($HdppmO_2$)$_3$]** — S7 – S8

4) IR spectra of ligand and complex **1** — S8 – S9

5) ^{1}H and ^{31}P{^{1}H} NMR spectra of compound **1** — S9 – S10

6) HRMS of compound **1** — S10 – S11

7) Variable temperature (VT) ^{1}H and ^{31}P{^{1}H} NMR spectra — S11 – S12

8) ^{19}F{^{1}H} NMR spectrum of reaction mixture involving redox transmetallation protolysis for synthesis of compound **1** — S13

9) ^{19}F{^{1}H} and ^{31}P{^{1}H} spectrum of reaction mixture involving redox protolysis. — S14

10) HRMS of compound **2** — S15

11) Crystallographic data — S15 – S18

12) References — S18 – S19

*S1.1: Synthesis of compound **1** and monitoring of the consumption of reactants by ^{1}H NMR and $^{31}P\{^{1}H\}$ NMR spectroscopy*

S1.1.1 Reaction between [YbCp$_2$(dme)]:H$_2$ dppmO$_2$ mole ratio 1:1

a) [YbCp$_2$(dme)] (100 mg, 0.25 mmol) and methylenebis(diphenylphosphane) dioxide (H$_2$dppmO$_2$) (96 mg, 0.23 mmol) in THF (5 ml) at room temperature, was stirred for 30 minutes. Unreacted [YbCp$_2$(DME)]was present in the reaction mixture.

b) [YbCp$_2$(dme)] (10 mg, 0.025 mmol) and methylenebis(diphenylphosphane) dioxide (H$_2$dppmO$_2$) (9.6 mg, 0.023 mmol) in d_8-THF (1.5 ml) at room temperature, were stirred for 30 minutes. Unreacted [YbCp$_2$(DME)]was present in the reaction mixture. ^{1}H NMR of the reaction mixture (400 MHz, THF) δ 6.48 (s, 2H, *CH* (CpH)), 6.38 (d, *J* = 5.1 Hz, 2H, *CH* (CpH), 2.91 (s, 2H, *CH* (CpH), 5.68 (s, C$_5$H$_5$([Yb Cp$_2$ (dme)])). $^{31}P\{^{1}H\}$ NMR of the reaction mixture (162 MHz, THF) δ 39.06. (Corresponding to compound **1**)

S1.1.2 [YbCp$_2$(dme)]:H$_2$dppmO$_2$ mole ratio 1:3

a) [YbCp$_2$(dme)] (31.5 mg, 0.080 mmol) and H$_2$dppmO$_2$ (100 mg, 0.240 mmol) in dry THF (5 ml) at room temperature, were stirred for 30 minutes. Complete consumption of [YbCp$_2$(DME)]was observed in the reaction mixture.

b) [YbCp$_2$(dme)] (7.5 mg, 0.019 mmol) and H$_2$dppmO$_2$ (23.9 mg, 0.057 mmol) in d_8-toluene at room temperature, were stirred for 30 minutes. Complete consumption of Cp$_2$Yb(dme) was observed. ^{1}H NMR of the reaction mixture (400 MHz, C$_7$D$_8$) δ 6.45 (s, 2H), 6.27 (d, *J* = 4.8 Hz, 2H), 2.68 (s, 2H). $^{31}P\{^{1}H\}$ NMR (162 MHz, C$_7$D$_8$) δ 39.16. (Compound **1**)

1) Spectral Data

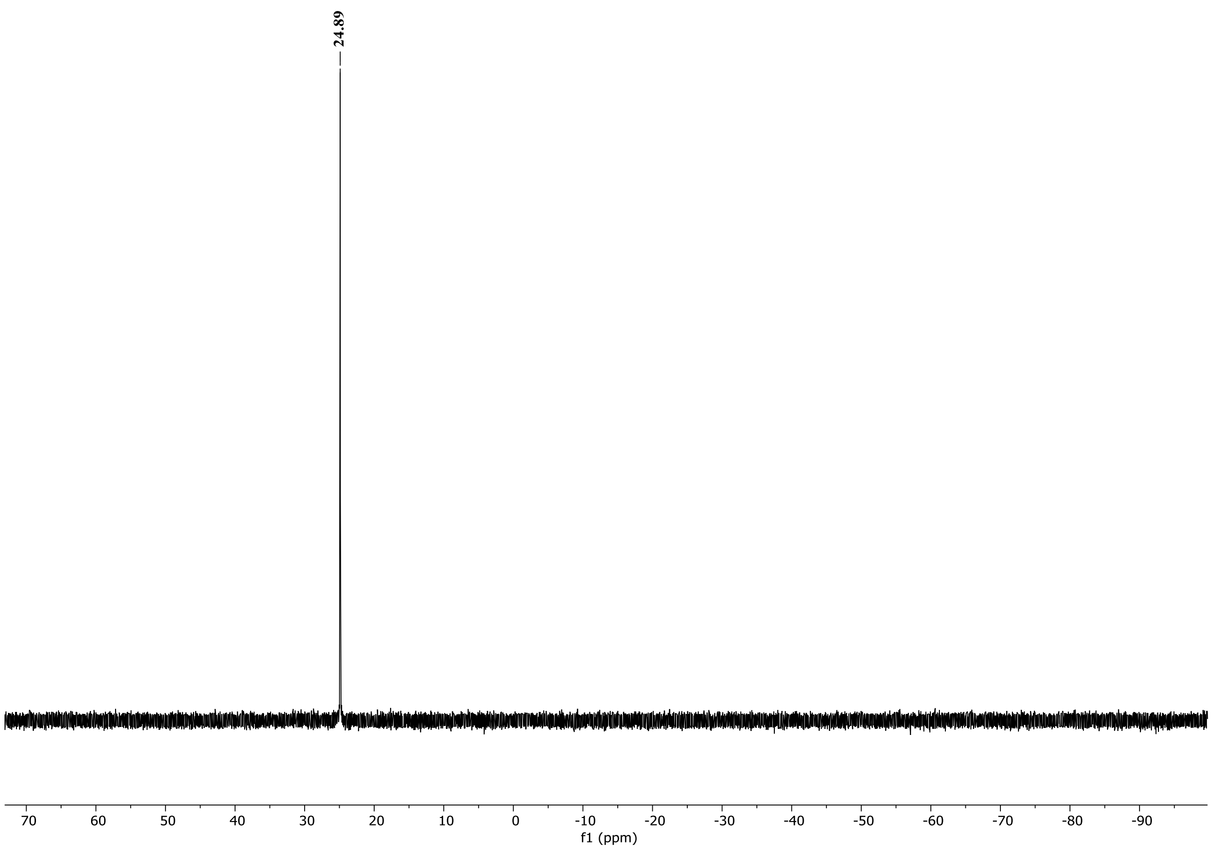

Figure S1a. $^{31}P\{^{1}H\}$ NMR spectrum of H$_2$dppmO$_2$ in CDCl$_3$ at 25 °C.

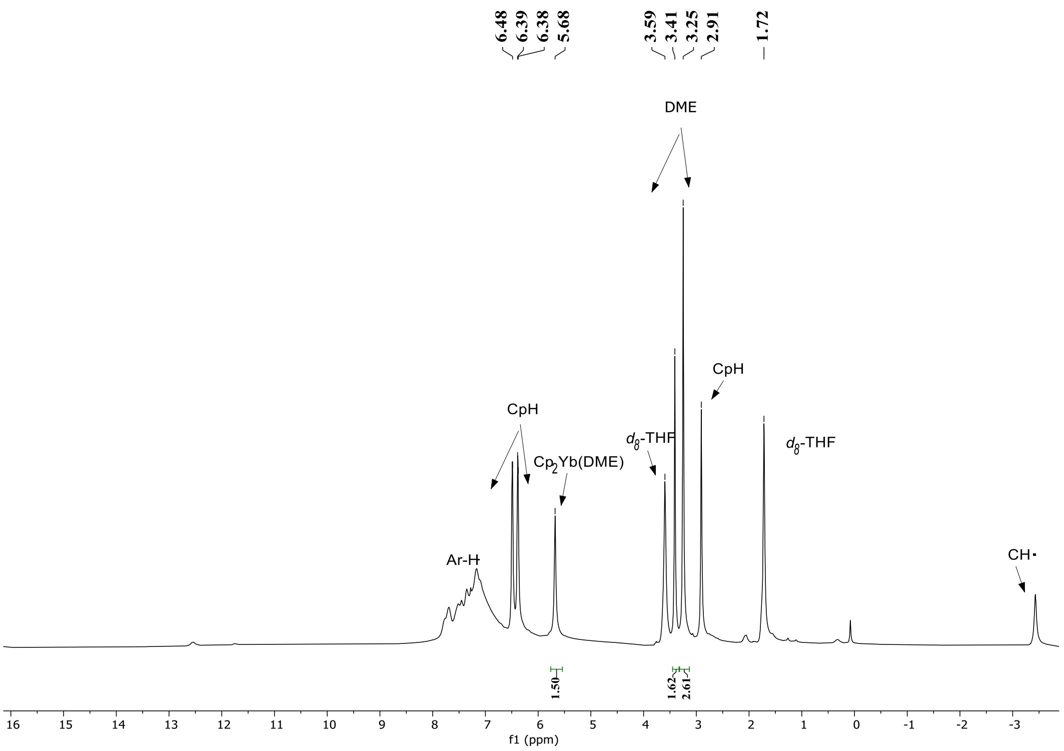

Figure S1b. ^{1}H NMR spectrum of the reaction mixture with 1:1 [$YbCp_2(DME)$] and H_2dppmO_2 in d_8-THF at 25 °C.

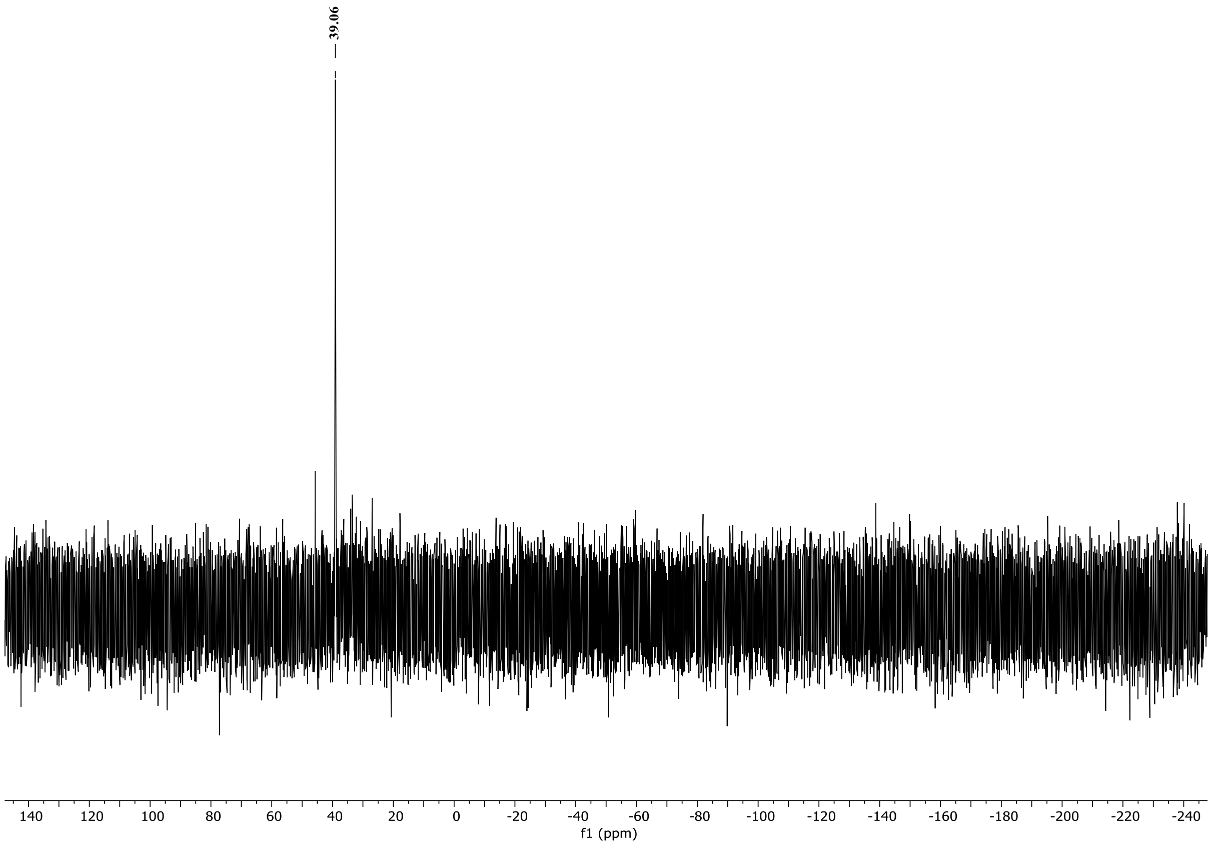

Figure S1c. $^{31}P\{^{1}H\}$ NMR spectrum of the reaction mixture with 1:1 [$YbCp_2$(DME)] and H_2dppmO_2 in d_8-THF at 25 °C.

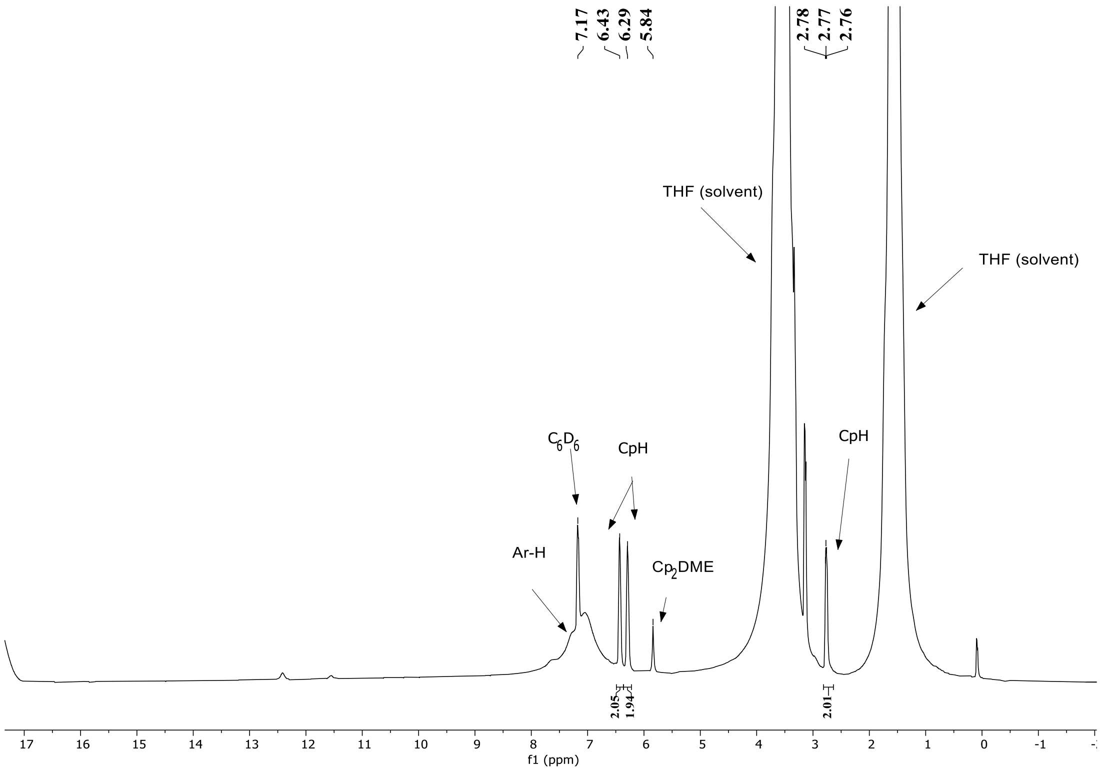

Figure S1d. ^{1}H NMR spectrum of the reaction mixture (in THF) with 1:2 [$YbCp_2$(DME)] and H_2dppmO_2 in d_6-C_6D_6 at 25 °C

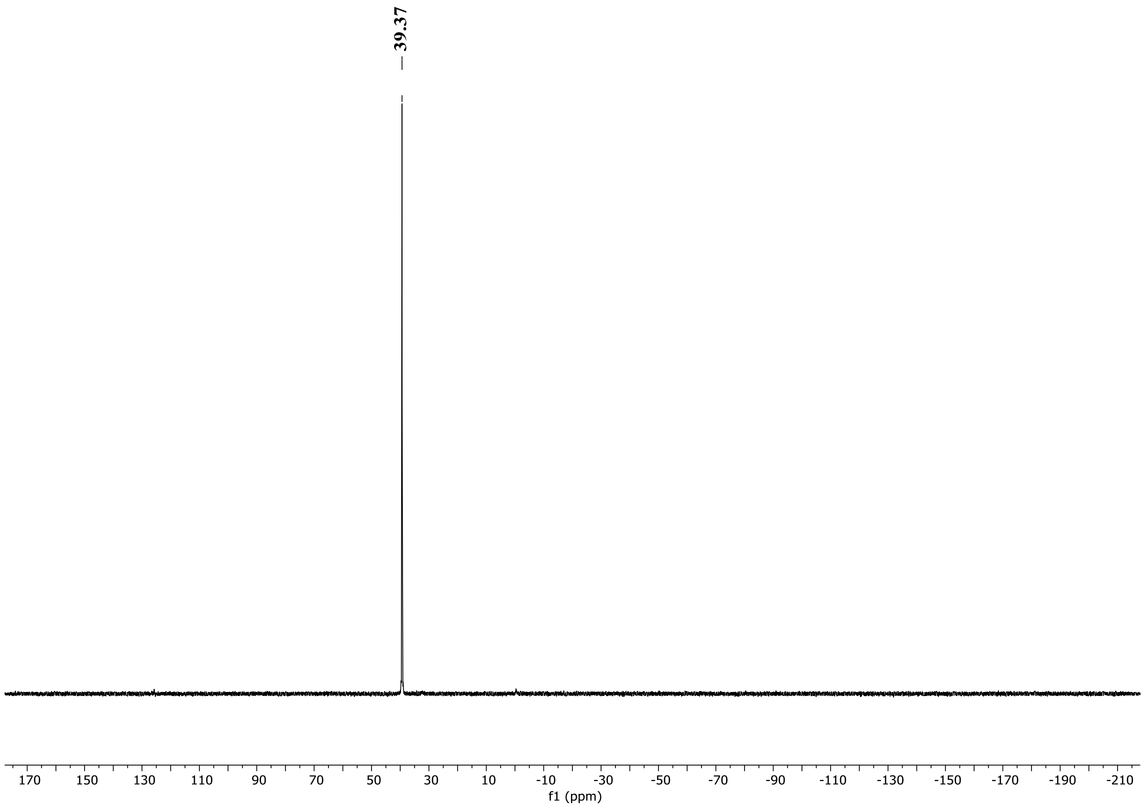

Figure S1e. $^{31}P\{^{1}H\}$ NMR spectrum of the reaction mixture (in THF) with 1:2 $[YbCp_2(DME)]$ and H_2dppmO_2 in d_6-C_6D_6 at 25 °C.

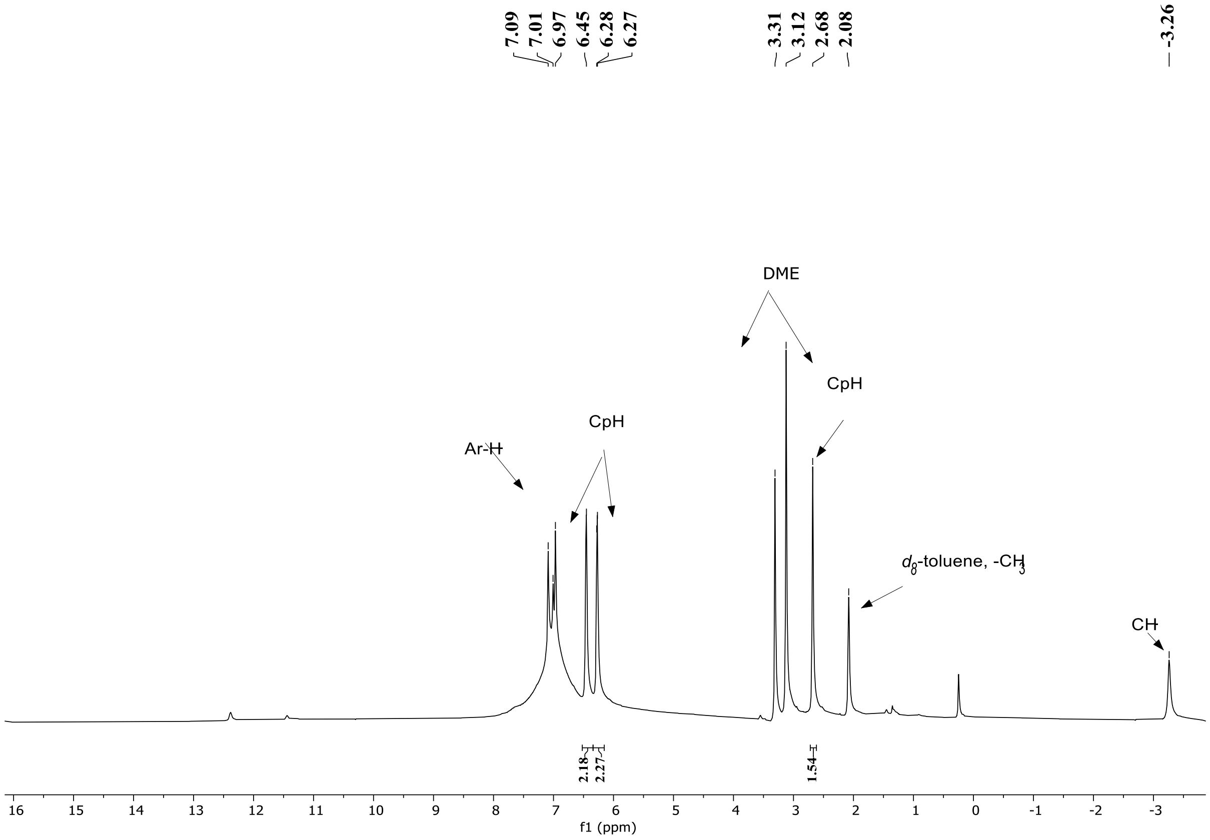

Figure S1f. ^{1}H NMR spectrum of the reaction mixture with 1:3 $[YbCp_2(DME)]$ and H_2dppmO_2 in d_8-toluene at 25 °C.

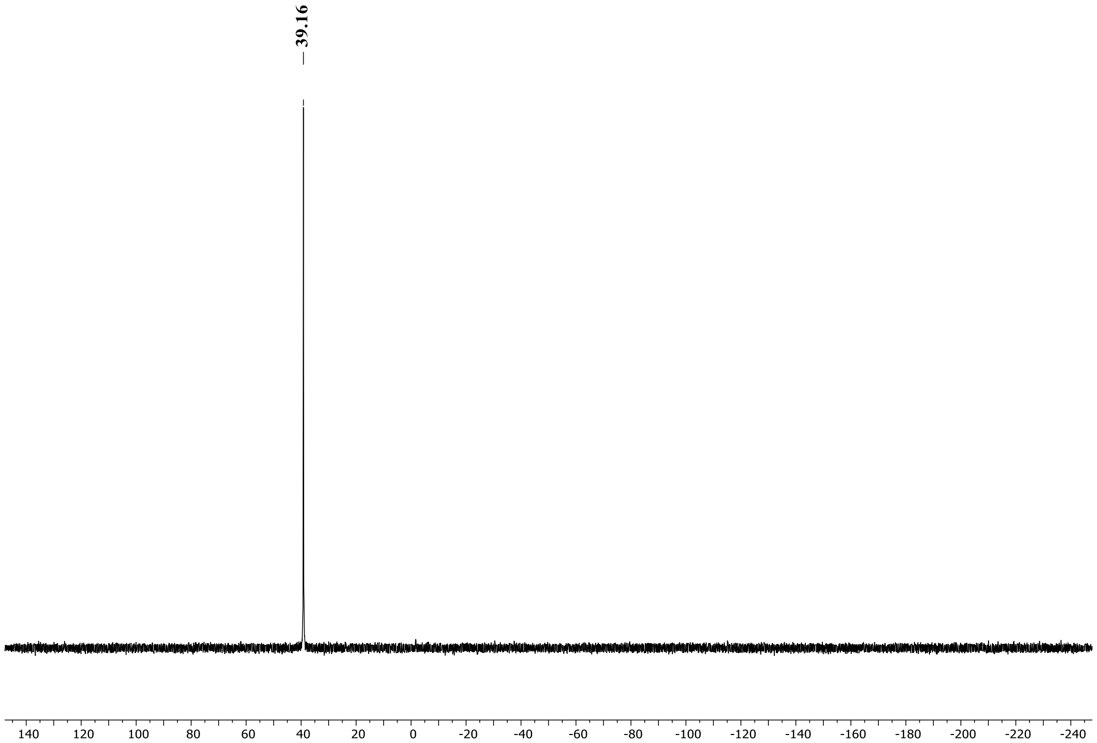

Figure S1g. $^{31}P\{^1H\}$ NMR spectrum of the reaction mixture with 1:3 [$YbCp_2$(DME)] and H_2dppmO_2 in d_8-toluene at 25 °C.

Table S1. Optimisation of stoichiometric ratio for the preparation of complex 1 (Figure S1)

Cp_2Yb(DME)(eq)	H_2dppmO_2 (eq)	^1H NMR (δ for Cp_2Yb(DME))	^1H NMR (δ for CpH)	$^{31}P\{^1H\}$ NMR (δ)
1	1	5.68	2.91(CH_2), 6.38-6.48 (4 -CH)	39.1
1	2	5.82	2.74(CH_2), 6.27-6.41 (4 -CH)	39.4
1	3	No peak	2.68(CH_2), 6.27-6.45 (4 -CH)	39.2
				24.9 (H_2dppmO_2)

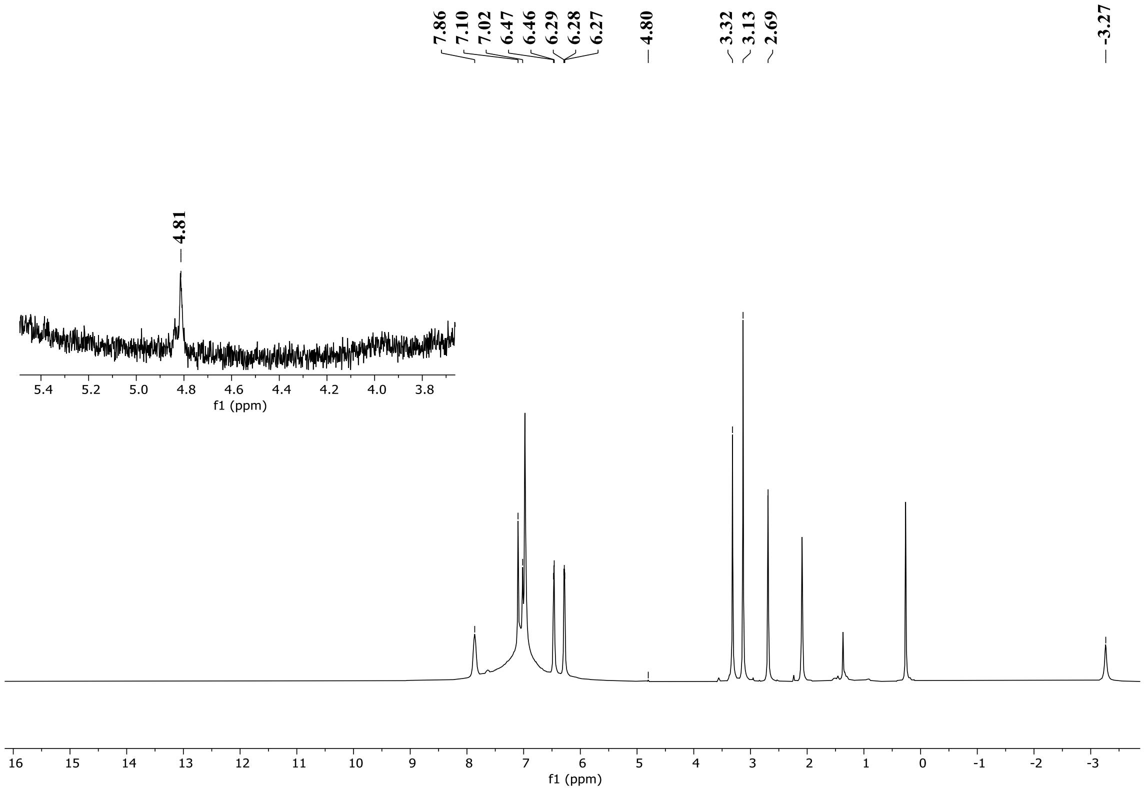

Figure S2a ^1H NMR at 25 °C of the reaction of [$YbCp_2$(DME)] and H_2dppmO_2 (1 : 3) in d_8-toluene showing evolution of H_2 gas in the reaction mixture.

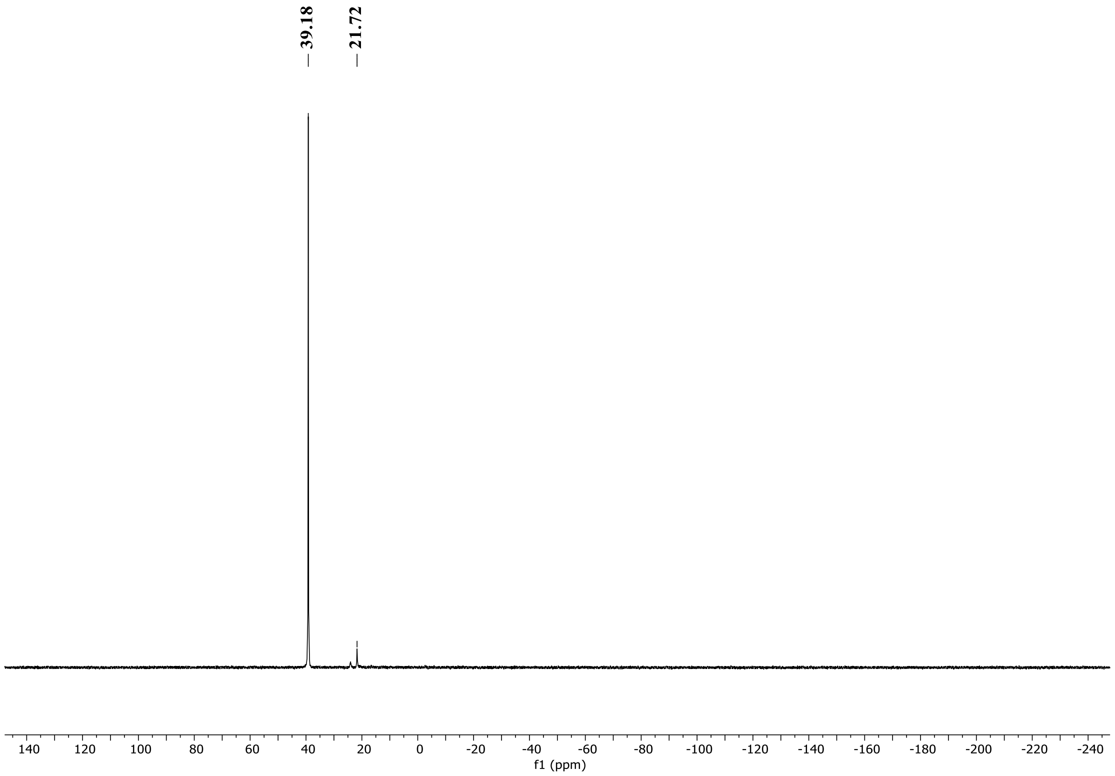

Figure S2b $^{31}P\{^1H\}$ at 25 °C of the reaction of [YbCp$_2$(DME)] and H$_2$dppmO$_2$ (1 : 3) in *d$_8$*-toluene showing evolution of H_2 gas in the reaction mixture.

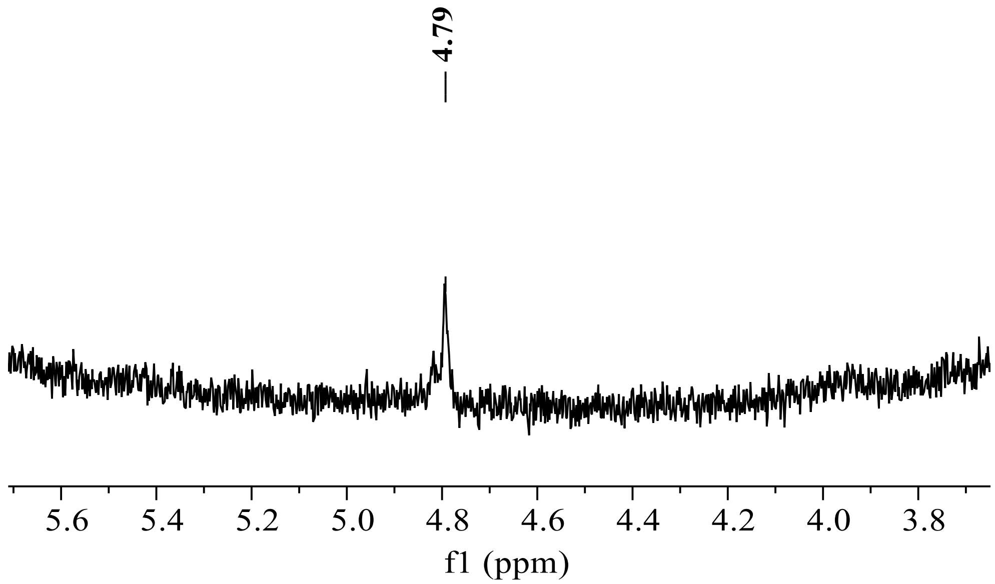

Fig S2c ^1H NMR at 25 °C of the reaction of [YbCp$_2$(DME)] and H$_2$dppmO$_2$ (1 : 3) in *d$_8$*-toluene showing evolution of H_2 gas in the reaction mixture.

Table S2. Averaged natural charges of Yb(III) and OPCPO for **[Yb(HdppmO$_2$)$_3$]**

All calculations reported were performed using the Gaussian 09 suite of programs. Single point calculations were performed on complex **1** using the basis set cc-pVDZ-DK3[1-4]/6-31G (d, p)[2-6] for carrying out natural bond analysis and chosen B3PW91 functional for our study. The cc-pVDZ-DK3 is

basis set used for Yb metal and 6-31G (d, p) basis set is used for C, H, O and P. Natural Bond Orbital (NBO) analysis was carried out on complex **1.**

Complex	q_{Yb}	q_{O}	q_{P}	q_{CH}	$q_{C\text{-}Ar}$
**1**	1.455	1.080	2.048	-1.431	-0.418

q is the average natural charge

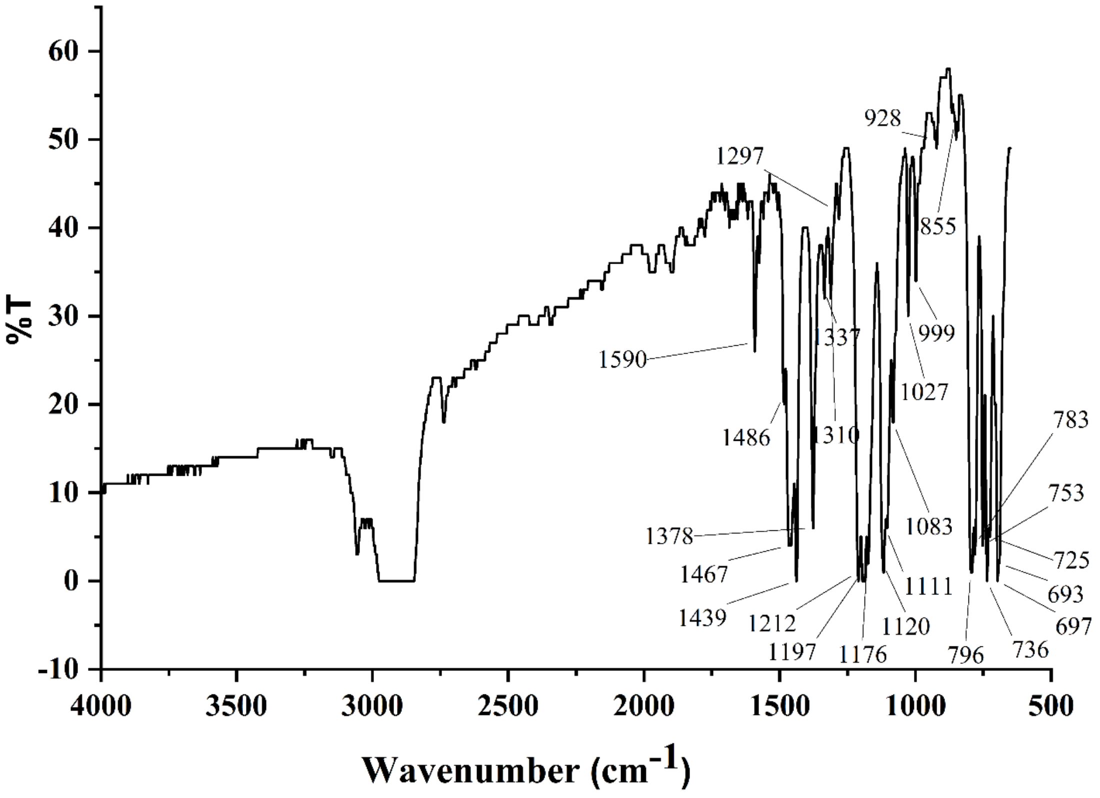

Figure S3. IR(Nujol) spectrum of H_2dppmO_2.

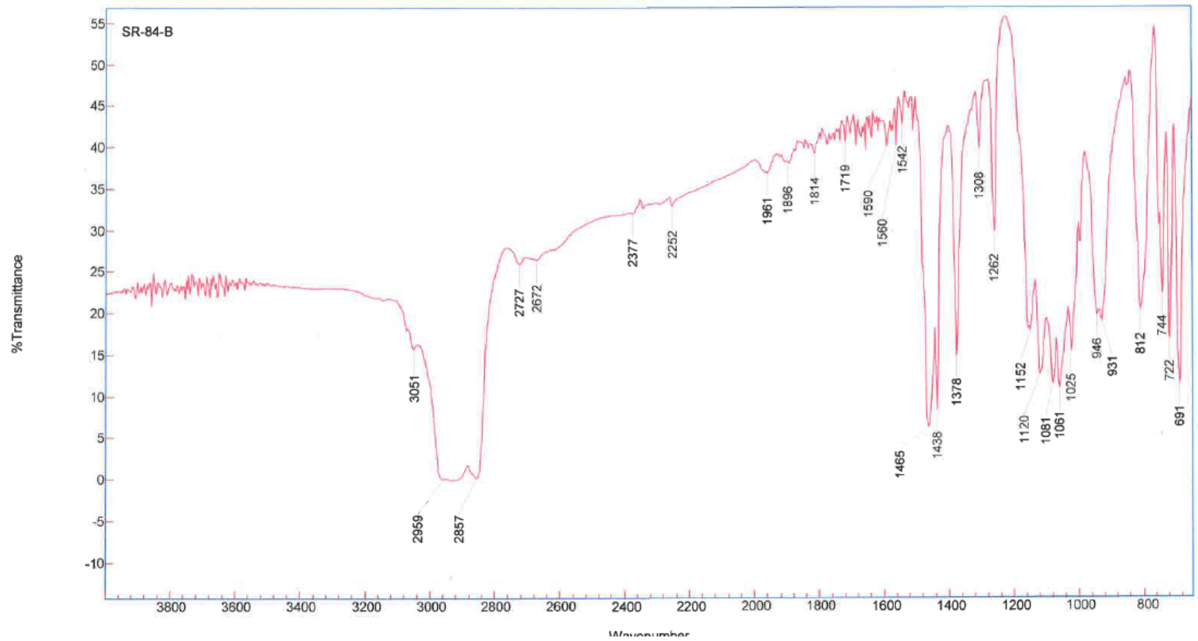

Figure S4. IR(Nujol) spectrum of [Yb($HdppmO_2$)$_3$](**1**).

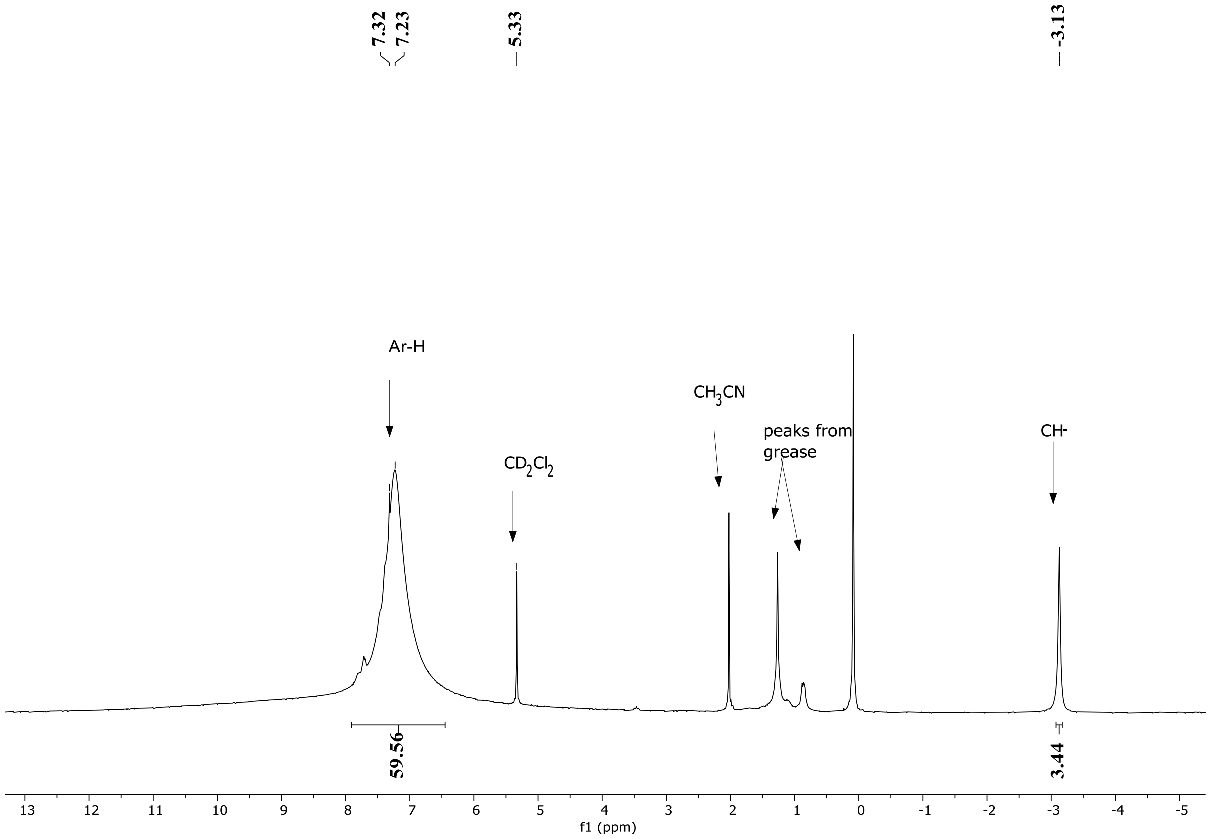

Figure S5a. ^{1}H NMR spectrum of compound **1** in d_2-CD_2Cl_2 at 25 °C.

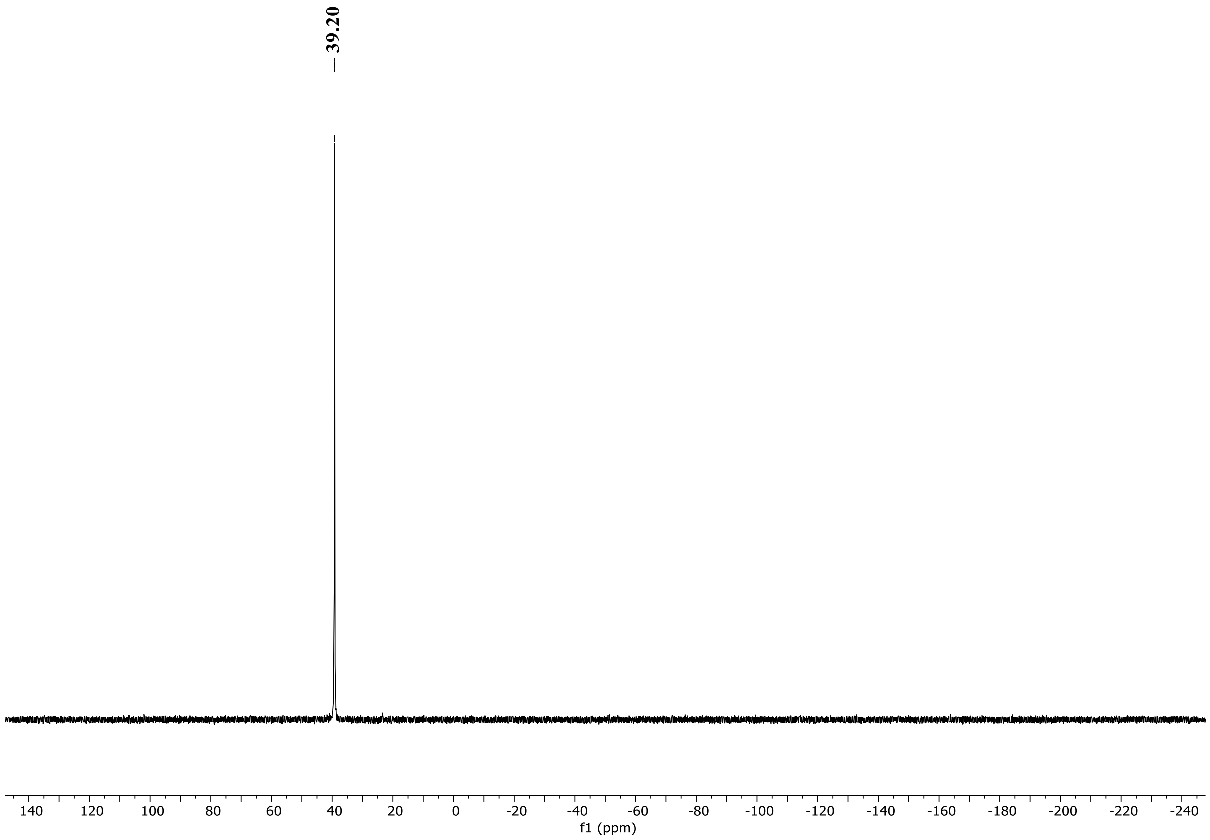

Figure S5b. $^{31}P\{^{1}H\}$ NMR spectrum of compound **1** in d_2-CD_2Cl_2 at 25 °C.

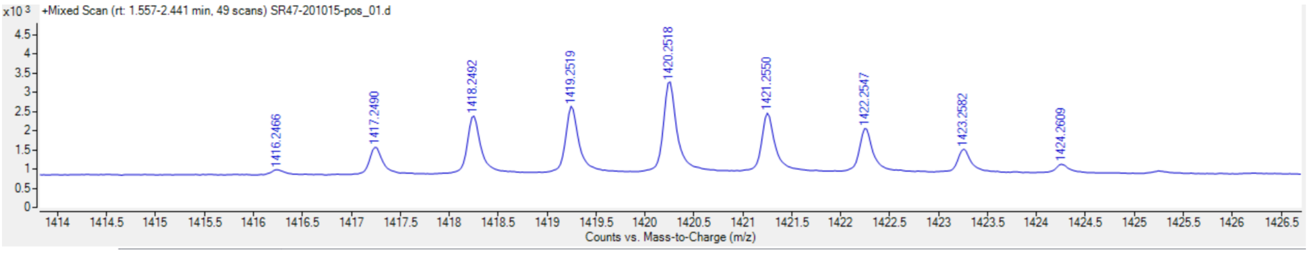

Figure S6a. HRMS spectrum of [Yb($HdppmO_2$)$_3$] (found).

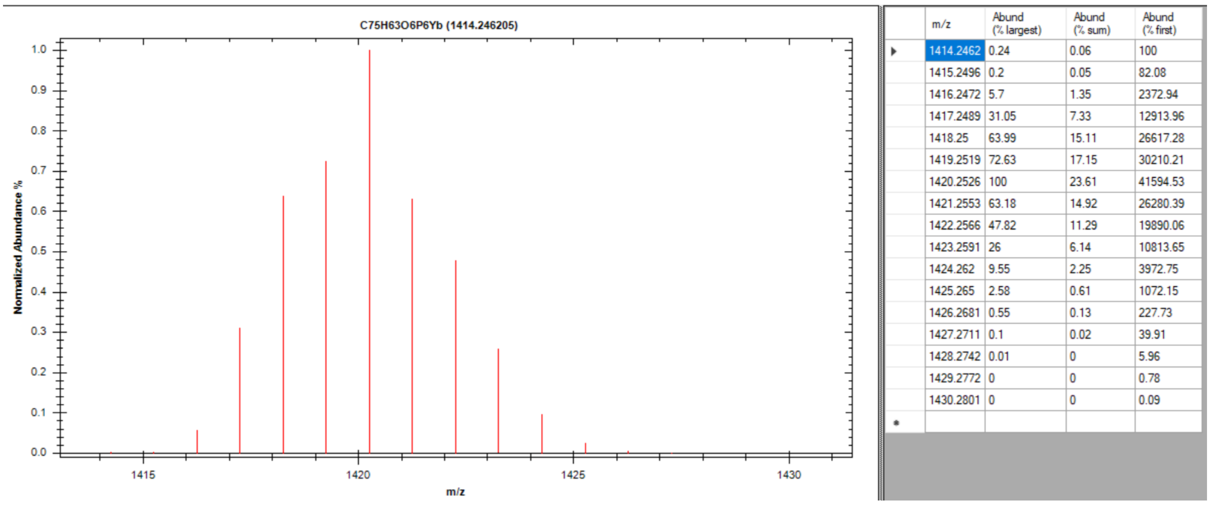

m/z	Abund (% largest)	Abund (% sum)	Abund (% first)
1414.2462	0.24	0.06	100
1415.2496	0.2	0.05	82.08
1416.2472	5.7	1.35	2372.94
1417.2489	31.05	7.33	12913.96
1418.25	63.99	15.11	26617.28
1419.2519	72.63	17.15	30210.21
1420.2526	100	23.61	41594.53
1421.2553	63.18	14.92	26280.39
1422.2566	47.82	11.29	19890.06
1423.2591	26	6.14	10813.65
1424.262	9.55	2.25	3972.75
1425.265	2.58	0.61	1072.15
1426.2681	0.55	0.13	227.73
1427.2711	0.1	0.02	39.91
1428.2742	0.01	0	5.96
1429.2772	0	0	0.78
1430.2801	0	0	0.09

Figure S6b. HRMS spectrum of [Yb(HdppmO$_2$)$_3$]. (calculated)

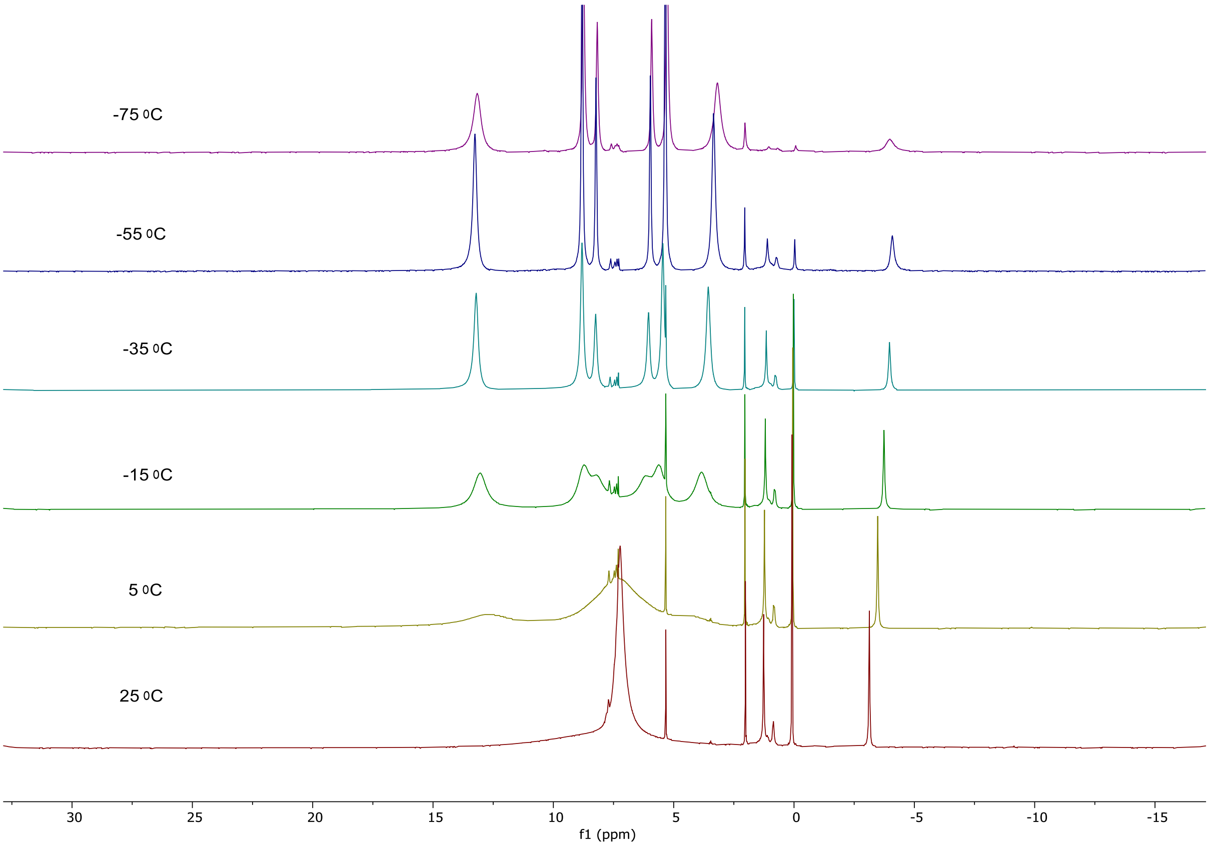

Figure S7a Variable temperature ^{1}H NMR spectrum of compound **1** in CD_2Cl_2.

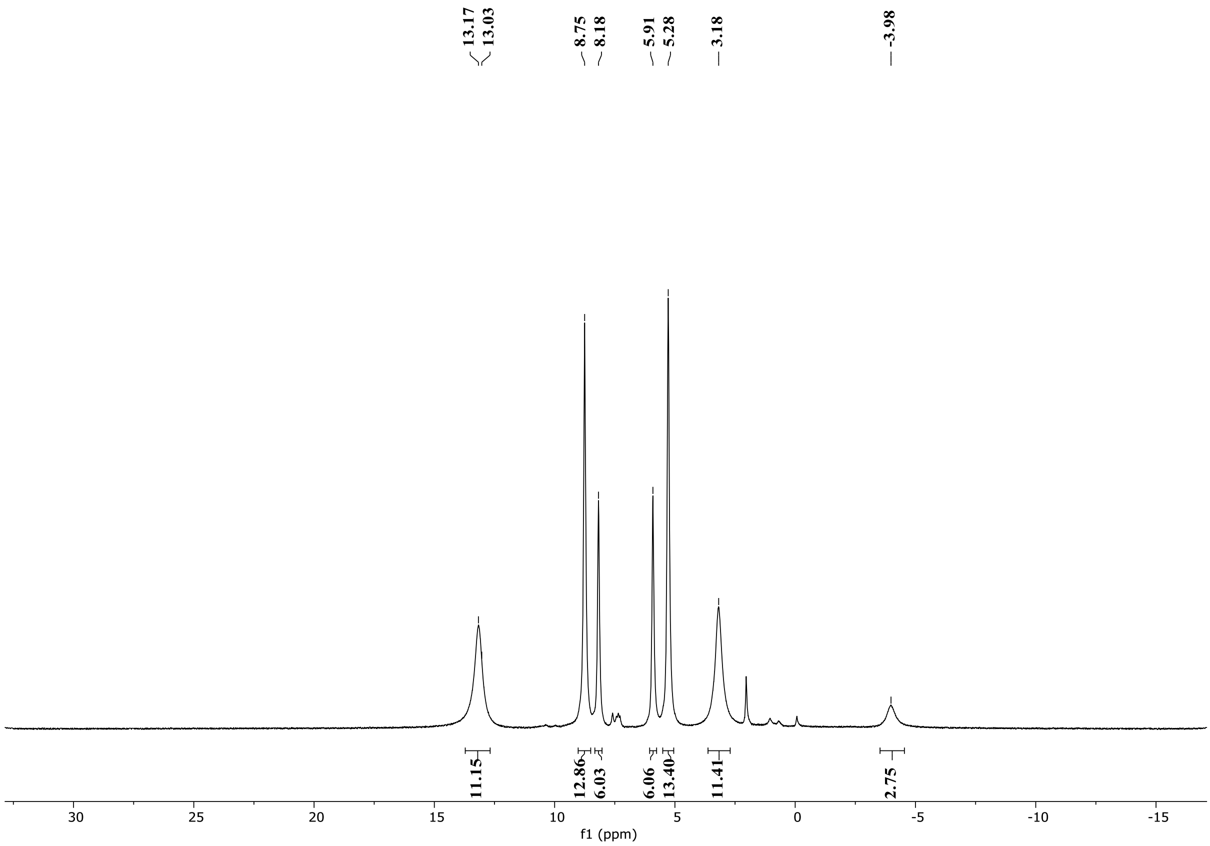

Figure S7b ^{1}H NMR spectrum of compound **1** in CD_2Cl_2 at -55 ^{o}C.

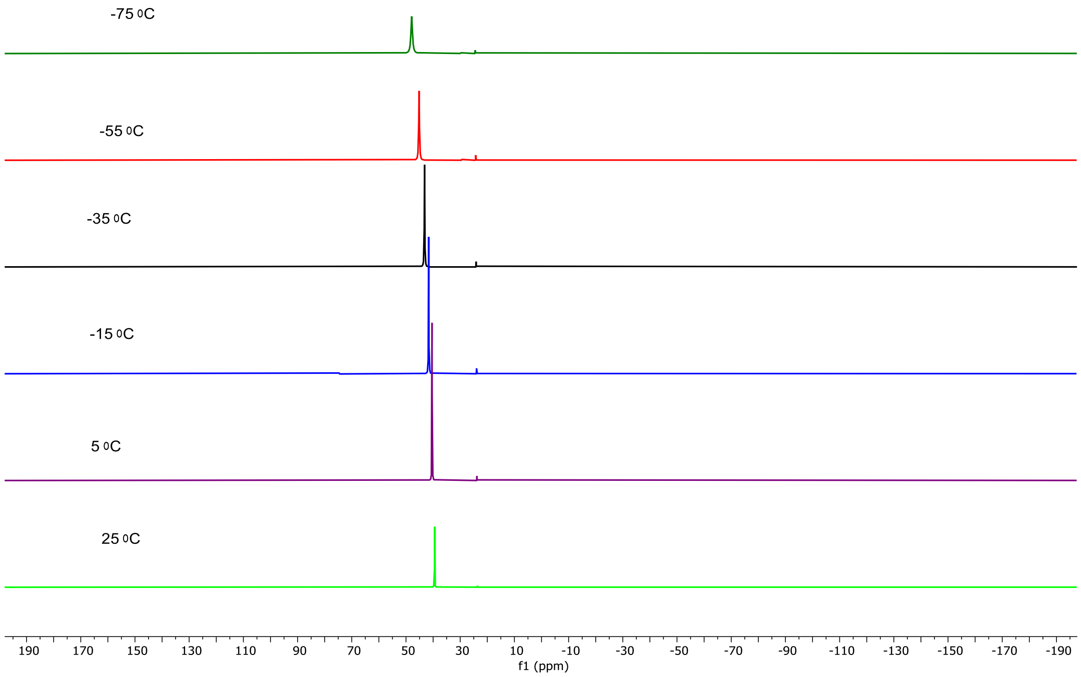

Figure S7c Variable temperature $^{31}P\{^{1}H\}$ NMR spectrum of compound **1** in CD_2Cl_2.

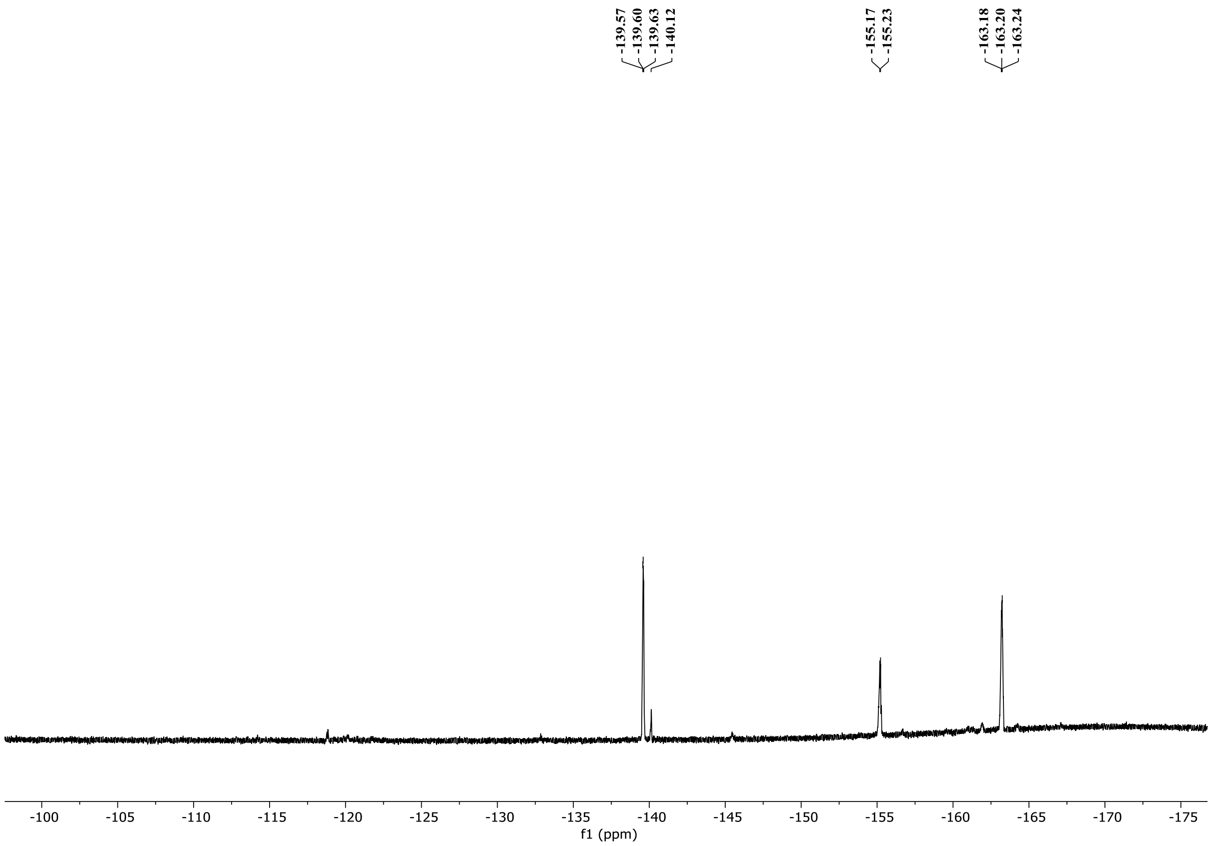

Figure S8 $^{19}F\{^{1}H\}$ NMR spectrum of reaction mixture involving redox transmetallation protolysis of H_2dppmO_2 with Yb and $Hg(C_6F_5)_2$ in C_6D_6 at 25 °C.

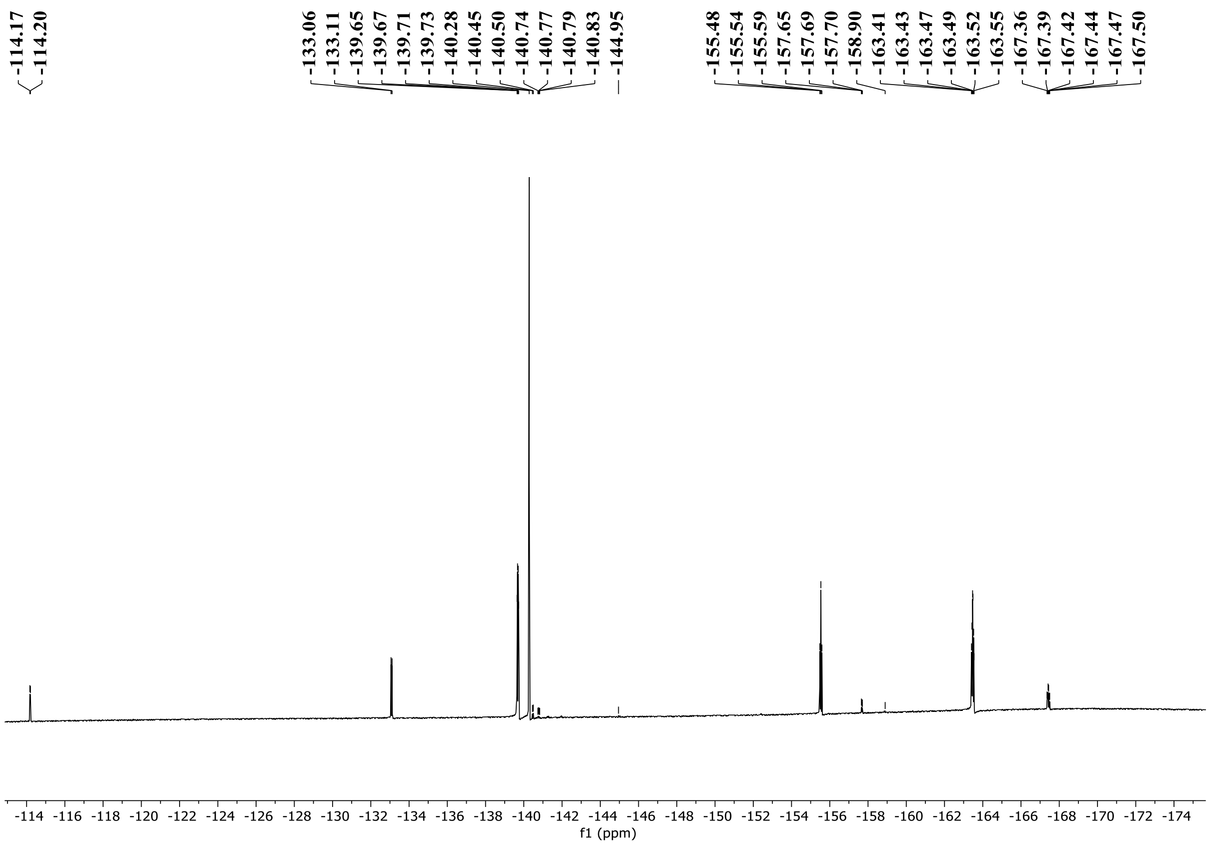

Figure S9a 19F NMR spectrum of reaction mixture involving redox protolysis of [Yb(C_6F_5)$_2$] with H_2dppmO_2 at 25 °C.

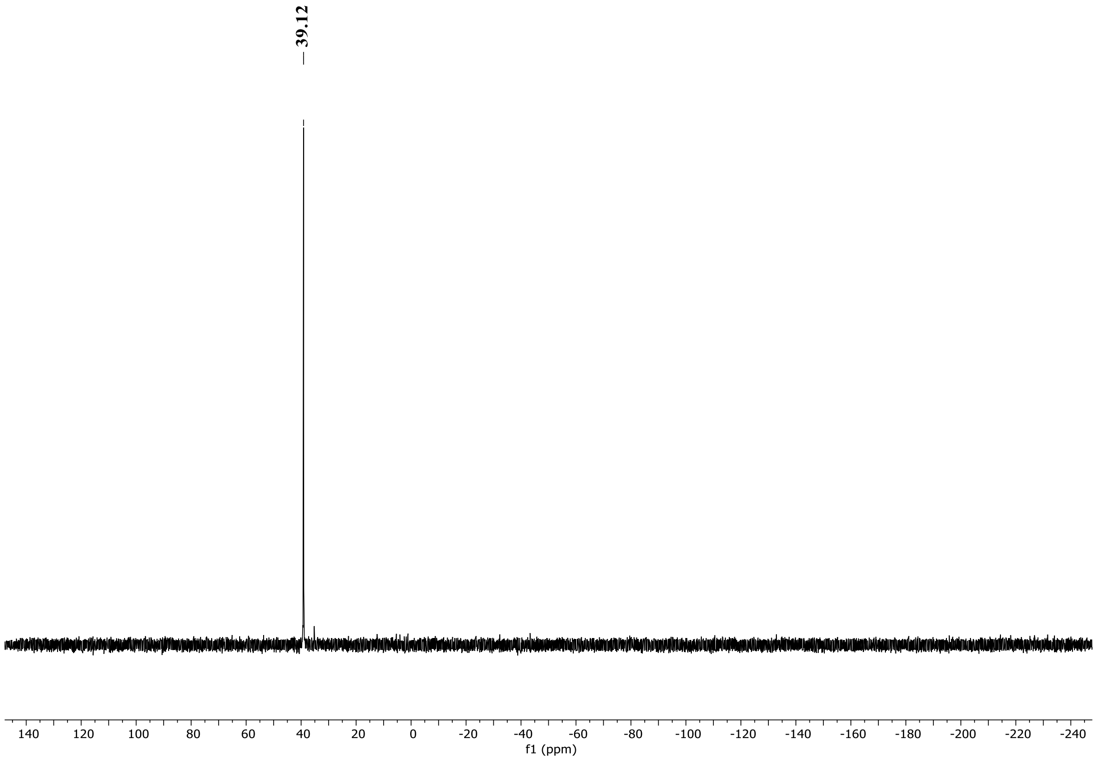

Figure S9b $^{31}P\{^1H\}$ NMR spectrum of reaction mixture involving redox protolysis of [Yb(C_6F_5)$_2$] with H_2dppmO_2 in C_6D_6 at 25 °C.

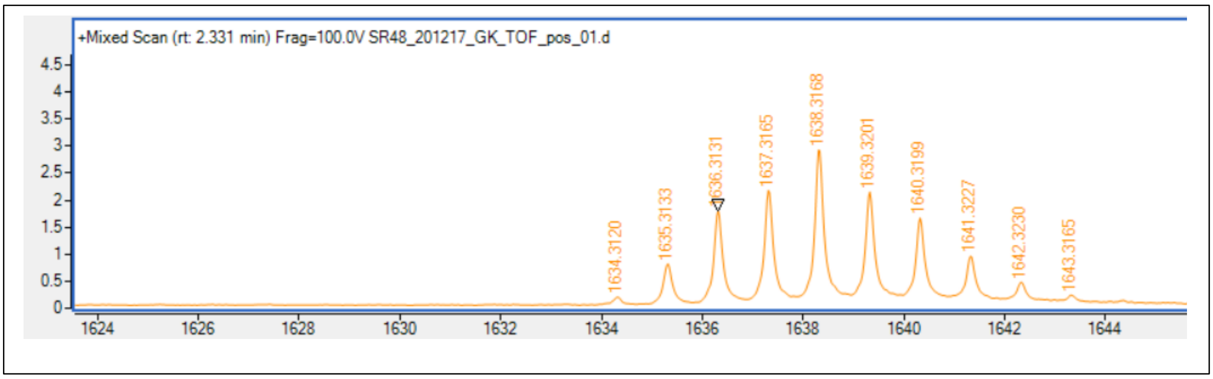

Figure S10a. HRMS of [Yb(HdppmO$_2$)$_3$] + Ph$_2$P(O)OH + H for compound [Yb$_4$(HddpmO$_2$)$_6$F$_6$] **2** , found.

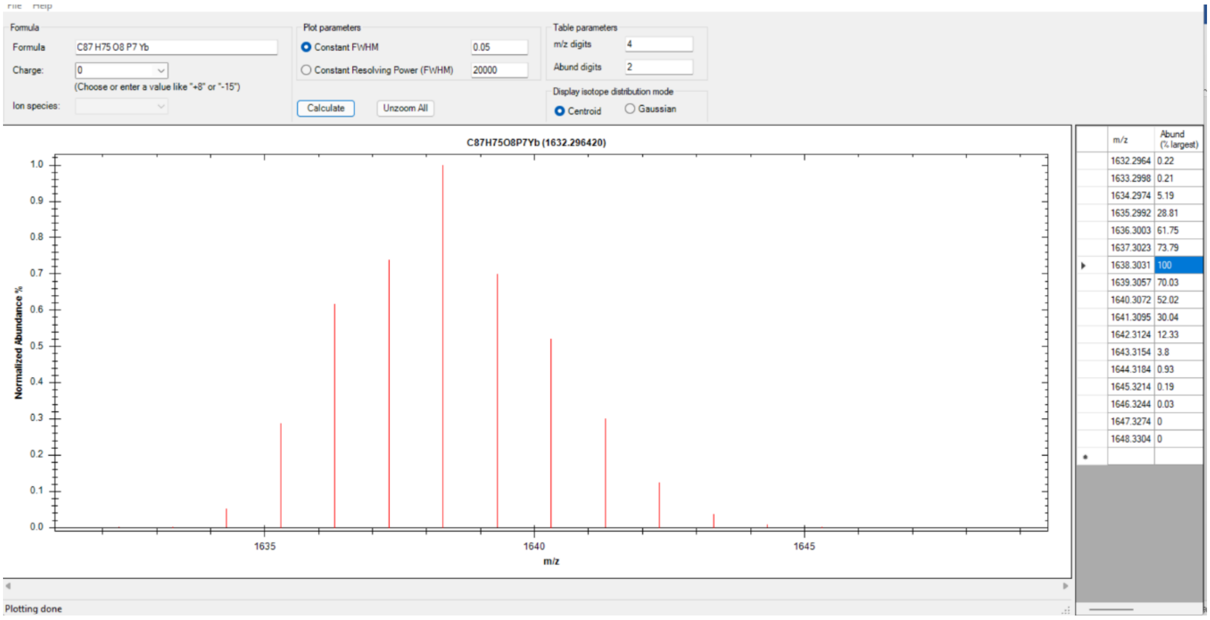

Figure S10b. HRMS of [Yb(HdppmO$_2$)$_3$] + Ph$_2$P(O)OH + H for compound [Yb$_4$(HddpmO$_2$)$_6$F$_6$] **2** , calculated.

2) Crystallographic Data

Crystals for X-ray structure analysis were grown using saturated solutions of acetonitrile (**1**), THF-C$_6$D$_6$ (**2**). Crystals were immersed in crystallography oil, and were measured on a BrukerX8 APEXIISCXRD or the MX1beamlines at the Australian Synchrotron. The structures were solved using Olex2[7] with the ShelXT[8] structure solution program using intrinsic phasing and refined with the SHELXL[9] refinement package using least-squares minimization. All atoms were refined anisotropically. Further details regarding the refinement and crystallographic data are listed in Table S3.

Table S3. Crystallographic data for compounds **1** and **2**.

Compound	**1**	**2**
Empirical formula	$[C_{77}H_{66}NO_6P_6Yb]CH_3CN$	$[C_{150}H_{126}F_6O_{12}P_{12}Yb_4]3(C_6D_6).6THF$
CCDC	2209922	2209923
Formula weight	1460.16	3965.24
Temperature/K	123.0	123.0
Crystal system	Triclinic	monoclinic
Space group	P-1	C2/c
a/Å	11.96594(7)	25.69430(10)
b/Å	13.72489(9)	26.50530(10)
c/Å	23.47857(10)	28.28910(10)
α/°	82.5315(5)	90
β/°	78.4653(5)	100.1070(10)
γ/°	64.8633(6)	90
Volume/Å^3	3415.84(4)	18966.89(14)
Z	2	4
ρ_{calc}g/cm^3	1.420	1.389
μ/mm^{-1}	4.288	4.986
F(000)	1486.0	8008.0
CuKα radiation,◎λ/ Å	1.54184	1.54184
Crystal size/mm^3	0.2 × 0.2 × 0.03	0.275 × 0.2 × 0.15
2θ range for data collection/°	7.124 to 155.01	7.474 to 154.956
Reflections collected	69732	143201
Independent reflections	14270 [R_{int} = 0.0541]	19914 [R_{int} = 0.0507, R_{sigma} = 0.0266]
Data/restraints/parameters	14270/0/821	19914/191/1113
Goodness-of-fit on F^2	1.079	1.067
R_1[a]	0.0330	0.0413
wR_2[b]	0.0877	0.1204

[a] R1 = Σ(||F0|-|Fc||)/Σ|F0|,F0> 4s(F0). [b]ωR2 = {Σ[w(F02-Fc2)2/Σ[w(F02)2]}1/2

Table S4. Selected bond lengths (Å) and angles (°) for compounds **1** and **2**.

**S.No.**	Bond length	(Å)	Bond angle	(°)
**1**	Yb1-O1	2.2263(15)	P2-C13-P1	117.15(13)
	Yb1-O2	2.2033(16)	P3-C38-P4	117.27(13)1
	Yb1-O3	2.2221(15)	P5-C63-P6	119.03(13)
	Yb1-O4	2.2319(14)	O6-Yb1-O2	175.08(6)

	Yb1-O5	2.1970(16)	O6-Yb1-O3	92.47(6)
	Yb1-O6	2.1953(16)	O6-Yb1-O4	87.63(6)
	P1-O1	1.5317(16)	O6-Yb1-O5	85.01(6)
	P2-O2	1.5299(16)	O6-Yb1-O1	92.63(6)
	P3-O3	1.5274(16)	O2-Yb1-O3	92.10(6)
	P4-O4	1.5152(16)	O2-Yb1-O4	90.90(6)
	P5-O5	1.5183(17)	O2-Yb1-O1	89.32(6)
	P6-O6	1.5201(17)	O3-Yb1-O4	85.35(6)
	P1-C13	1.715(2)	O3-Yb1-O1	88.76(6)
	P2-C13	1.714(2)	O5-Yb1-O2	90.36(6)
	P3-C38	1.706(2)	O5-Yb1-O3	176.86(6)
	P4-C38	1.711(2)	O5-Yb1-O4	92.66(6)
	P5-C63	1.716(2)	O5-Yb1-O1	93.23(6)
	P6-C63	1.717(2)	O1-Yb1-O4	174.10(6)
**2**	Yb1-F1	2.1370(3)	F4-Yb1-F3	73.56(7)
	Yb1-F3	2.1976(18)	F1-Yb1-F4	91.25(9)
	Yb1-F4	2.1854(18)	F1-Yb1-F3	83.50(7)
	Yb2-F2	2.16282(18)	F1-Yb1-O2	176.11(7)
	Yb2-F3	2.1845(19)	F1-Yb1-O1	87.47(8)
	Yb2-F4	2.1824(18)	F1-Yb1-O6[1]	90.81(9)
	Yb1-O1	2.167(2)	O2-Yb1-F4	87.88(8)
	Yb1-O2	2.182(2)	O2-Yb1-F3	99.88(8)
	Yb2-O3	2.189(2)	O1-Yb1-F4	92.33(8)
	Yb2-O4	2.162(2)	O1-Yb1-F3	163.00(9)
	P1-O1	1.515(2)	O1-Yb1-O2	88.78(9)
	P2-O2	1.518(2)	O6[1]-Yb1-F4	167.48(9)
	P3-O3	1.524(2)	O6[1]-Yb1-F3	94.42(9)
	P4-O4	1.520(2)	O6[1]-Yb1-O2	90.85(9)
	P5-O5	1.524(2)	O6[1]-Yb1-O1	100.09(10)

P6-O6	1.517(2)	F4-Yb2-F3	73.88(7)
P1-C13	1.702(4)	F4-Yb2-O3	91.51(8)
P6-C13	1.695(4)	F2-Yb2-F4	83.04(7)
P2-C38	1.702(4)	F2-Yb2-F3	94.11(8)
P3-C38	1.712(4)	F2-Yb2-O3	174.42(8)
P4-C63	1.702(4)	F2-Yb2-O5[1]	89.70(7)
P5-C63	1.700(4)	F3-Yb2-O3	85.53(8)
		O5[1]-Yb2-F4	165.95(8)
		O5[1]-Yb2-F3	94.77(8)
		O5[1]-Yb2-O3	95.88(9)
		O4-Yb2-F4	95.01(8)
		O4-Yb2-F2	89.39(8)
		O4-Yb2-F3	167.81(8)
		O4-Yb2-O3	89.85(9)
		O4-Yb2-O5[1]	96.93(9)
		Yb2-F4-Yb1	106.42(7)
		Yb2-F3-Yb1	105.92(7)
		Yb1-F1-Yb1[1]	171.14(16)
		Yb2-F2-Yb2[1]	176.15(14)
		P6-C13-P1	126.1(2)
		P2-C38-P3	124.5(2)
		P5-C63-P4	128.5(2)

References:

1. Lu, Q.; Peterson, K. A., Correlation consistent basis sets for lanthanides: The atoms La-Lu. *J. Chem. Phys.* **2016,** *145,* 054111.
2. Pritchard, B. P.; Altarawy, D.; Didier, B.; Gibsom, T. D.; Windus, T. L., A New Basis Set Exchange: An Open, Up-to-date Resource for the Molecular Sciences Community. *J. Chem. Inf. Model.* **2019,** *59,* 4814-4820.
3. Schuchardt, K. L.; Didier, B. T.; Elsethagen, T.; Sun, L.; Gurumoorthi, V.; Chase, J.; Li, J.; Windus, T. L., Basis Set Exchange: A Community Database for Computational Sciences. *J. Chem. Inf. Model.* **2007,** *47,* 1045-1052.

4. Feller, D., The role of databases in support of computational chemistry calculations. *J. Comput. Chem.* **1996,** *17*, 1571-1586.
5. Hariharan, P. C.; Pople, J. A., The influence of polarization functions on molecular orbital hydrogenation energies. *Theor. Chim. Acta* **1973,** *28*, 213-222.
6. Hehre, W. J.; Ditchfield, R.; Pople, J. A., Self-Consistent Molecular Orbital Methods. XII. Further Extensions of Gaussian-Type Basis Sets for Use in Molecular Orbital Studies of Organic Molecules. *J. Chem. Phys.* **1972,** *56*, 2257-2261.
7. Dolomanov, O. V.; Bourhis, L. J.; Gildea, R. J.; Howard, J. A. K.; Puschmann, H., OLEX2: a complete structure solution, refinement and analysis program. *J. Appl. Cryst.* **2009,** *42* (2), 339-341.
8. Sheldrick, G. M., SHELXT-Integrated space-group and crystal-structure determination. *Acta Crystallogr., Sect. A: Found. Crystallogr.* **2015,** *71* (1), 3-8.
9. Sheldrick, G., Crystal structure refinement with SHELXL. *Acta Crystallogr., Sect. C: Struct. Chem.* **2015,** *71* (1), 3-8.
